# Supplementary material for: Performance study of oil-injection hermetic CO2 scroll compressor for automotive air conditioning system
Source: PLoS One. 2024 Dec 6;19(12):e0311188. doi: 10.1371/journal.pone.0311188 (PMC11623558; doi:10.1371/journal.pone.0311188)
Supplement: S1 Table — (DOCX) [file pone.0311188.s001.docx]

**S1 Table. Performance index of prototype obtained in the research**

Experimental condition:

*P*_s_=4.5MPa, *P*_d_=10MPa

Experimental results:

| Different mass of oil to refrigerant | Volumetric efficiency  *η*_v_ | Indicated efficiency  *η*_v_ | Mass flow rate  *q*_m_ | Discharge temperature  *T*_d_ | Input power  *P*_in_ |
| --- | --- | --- | --- | --- | --- |
| 0 | 0.4459 | 0.383 | 72.848 | 95.44 | 1615.31 |
| 2 | 0.536 | 0.4441 | 85.236 | 90.19 | 1583.47 |
| 4 | 0.7043 | 0.5755 | 112.803 | 75.17 | 1514.35 |
| 6 | 0.6238 | 0.5093 | 98.68 | 82.31 | 1553.47 |
| 8 | 0.6783 | 0.5518 | 106.31 | 73.86 | 1526.91 |
